# A Bibliometric Analysis of the Impacts of Air Pollution on Children

**DOI:** 10.3390/ijerph17041277

**Published:** 2020-02-17

**Authors:** Jinfang Sun, Zhichao Zhou, Jing Huang, Guoxing Li

**Affiliations:** 1 Office of Epidemiology, Chinese Center for Disease Control and Prevention, Beijing 102206, China; sunjf@chinacdc.cn; 2 Peking University Health Science Library, Beijing 100191, China; zhouzc1987@bjmu.edu.cn; 3 Department of Occupational and Environmental Health Sciences, Peking University School of Public Health, Beijing 100191, China; jing_huang@bjmu.edu.cn

**Keywords:** air pollution, children, research trend, bibliometric study

## Abstract

In recent years, many researchers have investigated the association between air pollution and children. However, there has been little research to provide a macroscopic overview in this field. The aim of this study is to characterize the scientific production around the world in this area and map the trends. The relevant literature was searched from 1999 to 2018. To guarantee the quality of the literature, we combined the PubMed and WoS databases. The built-in statistics tools of the Web of Science website were used to display the trend of articles published by year and the distribution of journals. By CiteSpace (5.5.R2), the reference co-citation and burst keywords were extracted. In total, 15,999 target English documents were obtained. We summarized the characteristics of published documents, of research institutes’ cooperation, and of the contents. As part of a research hotspot, ten clusters are presented, four popular topics are elaborated. Twenty-four burst words were obtained and analyzed. China has received more attention in recent years. Researchers in this field could carry out more cohorts’ studies and fine particulate matter is one good air pollution index. Household air pollution exposure and children’s lung function should be paid more attention.

## 1. Introduction

Air pollution has become a global public health problem. Based on the global burden of disease (GBD) report, Ambient PM_2.5_ was the fifth-ranking mortality risk factor in 2015, outdoor air pollution has a number of adverse effects on human health [1,2]. Due to the fact that children’s body systems are still developing, they are more vulnerable than adults if exposed to harmful pollutants [3]. Plenty of results concerning the impact of air pollution on children have been reported [4]. Nevertheless, most of the literature focuses on specific diagnosis, intervention, and policies in the area. Little research has attempted to explore it from a macro perspective.

The associations between air pollution and children’s health have been studied in numerous studies [5,6,7]. Mansourian found that PM_10_ and SO_2_ concentrations had statistically increased the number of respiratory admissions of children in Isfahan, Iran [8]. Khaniabadi et al. suggested that children and other vulnerable groups should be protected to reduce the adverse health impact of air pollution [9]. Siddique et al. obtained a positive association between the PM_10_ level in Delhi’s air and the prevalence of lower respiratory tract symptoms [10]. This research confirmed the adverse effects of air pollution on children’s health.

Bibliometric analysis, a well-established research method in information and library science, has been commonly used for revealing research outputs [11]. Bibliometric analyzes are important tools to evaluate and quantify the growth of literature for a particular subject. The bibliometric method has been used in different contexts to investigate data showing increases in the number of publications and identification of the main authors, research institutions, and countries [12]. In this study, we retrieve the therapy-related papers of the last 20 years in the main bibliographic databases, analyze them from the perspective of literature publication, so as to reacquaint the transition and inheritance of the impacts of air pollution on children, and meanwhile reveal the possible research focuses for the future, which could help researchers in their topic selection in this field. Bibliometrics is a useful method to explore the most impactful authors, countries/regions, construct collaboration networks, and identify research key topics in particular areas. In the present study, a bibliometric analysis is conducted to (1) determine the research landscape of air pollution on children in terms of the year, journals, institutions, keywords, and references; (2) identify the cooperation among institutions; and (3) explore the hot topics and developments in the future.

## 2. Materials and Methods

### 2.1. Literature Sources

PubMed is the most comprehensive biomedical literature database worldwide, which is developed and maintained by the National Center for Biotechnology Information at the U.S. National Library of Medicine. PubMed comprises more than 28 million citations for biomedical literature from MEDLINE, life science journals, and online books. Hence, we chose PubMed as the English literature source. In order to search the literature about impacts of air pollution on children as comprehensively as possible, we performed literature retrieval both in PubMed and the Web of Science Core Collection on 23 June 2019.

### 2.2. Search Strategy

At the beginning, we found out whether the search word was covered by the Medical Subject Headings (MeSH) database, which is the National Library of Medicine’s controlled vocabulary thesaurus used for indexing articles for PubMed. If not, the word would be searched as a text word. For the concept of air pollution, we found several MeSH terms that are closely related to it, such as air pollution, air pollutants, and particulate matter. Next, some MeSH terms regarding the main monitoring component of air pollution was also included, such as Nitrogen Dioxide, Sulfur Dioxide, Ozone, Vehicle Emissions, and Carbon monoxide. Considering that the same concept may be expressed in different ways in scientific papers, we also searched the concept of air pollution above-mentioned in the text word. In addition, atmospheric pollution, inhalable particles, and inhalable particulate matter were also included in the search strategy as text words. According to the Convention on the Rights of the Child, issued by United Nations, a child means every human being below the age of eighteen years unless under the law applicable to the child, majority is attained earlier [13]. Therefore, for the concept of child in our research, the corresponding terms in the Mesh database are Infant, Child, and Adolescent, which are connected with logical operator “OR”, and we also add the Pediatrics as MeSH term. The logical link between the concepts of air pollution and child is “AND”, the search time is limited to the past two decades, which is 1999–2018, 12,156 target English documents were obtained.

**Figure 1:**
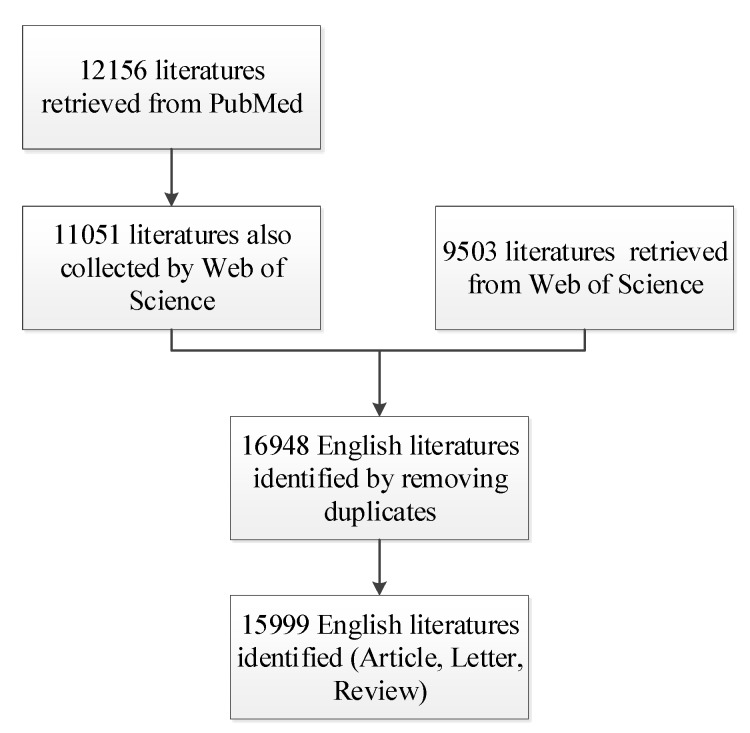
The flowchart of the research to search papers in databases.

Figure 1 shows the flowchart of our research. First, we got 12,156 target English documents through PubMed. PubMed is a comprehensive biomedical abstract database, but it cannot guarantee the quality of the literature, so we used the WoS database to again retrieve the literatures from the PubMed database and keep the high-quality literature. Then, we searched the literature about the impact of air pollution on children in the Web of Science database with the above keywords. In total, 9503 target English documents were obtained. Finally, we combined two datasets and just included the literature that are published in three types (article, review, letter) from 1999 to 2018, so we obtained 15,999 documents. The complete literature retrieval strategy is presented in Tables S1 and S2.

### 2.3. Data Visualization and Analysis

The identified articles were systematically analyzed by the Web of Science website and CiteSpace (5.5.R2) (https://sourceforge.net/projects/citespace/) [14]. The Web of Science website has built-in statistics tools to display the trend of articles published by year and the distribution of journals. CiteSpace has been continuously developed to meet the needs for visual analytic tasks of science mapping. CiteSpace takes a set of bibliographic records as its input and models the intellectual structure of the underlying domain in terms of a synthesized network on the basis of a time series of networks derived from each year’s publications. CiteSpace supports several types of bibliometric studies, including collaboration network analysis, co-word analysis, author co-citation analysis, document co-citation analysis, and text and geospatial visualizations. In this study, we focus on the document co-citation analysis within the period of time between 1999 and 2018 and mine research hotspots and further discover and predict research frontiers by detecting the occurrence of mutation words in topics, abstracts, and keywords.

## 3. Results

### 3.1. The Annual Trend of Global Publications

**Figure 2:**
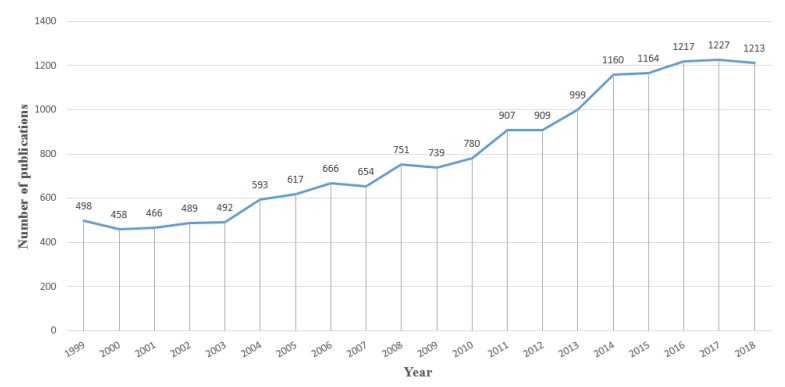
Global literature about air pollution and children published from 1999–2018.

Figure 2 showed the trend of publication number in the past twenty years. A total of 15,999 articles matched the retrieval criteria and were included for further analysis. The results indicated a consistently increasing trend from 498 articles in 1999 to 1213 articles in 2018.

### 3.2. Analysis of Core Journals

A total of 2052 scholarly journals have published articles regarding the research on the impact of air pollution on children. According to the Bradford law, literature on a topic is often concentrated in core journals. We usually chose the 10 or 20 academic journals with the most publications covering more than 30% of the articles for analysis, so as to make the results more representative [15,16,17,18].

The top 20 journals are presented in Table 1. The top 20 journals contributed 4885 (30.53%) articles. ENVIRONMENTAL HEALTH PERSPECTIVES (IF = 8.049) published the highest number of articles (596 articles, 3.73%), followed by ENVIRONMENTAL RESEARCH (IF = 5.026, 450 articles, 2.81%), SCIENCE OF THE TOTAL ENVIRONMENT (IF = 5.589, 377 articles, 2.36%), INTERNATIONAL JOURNAL OF ENVIRONMENTAL RESEARCH AND PUBLIC HEALTH (IF = 2.468, 233 articles, 2.01%). Among the top 20 journals, around 75% were from the United States (nine) and England (six).

### 3.3. Characteristics of the Institutions Contribution

Overall, 15,999 articles in research were published by 8425 institutions. Fifteen institutes were selected because of their critical role for scientific knowledge dissemination [19,20]. The list of top 15 institutions is presented in Table 2, which published about 24.81% of all the articles. Harvard University had the most publications (585), followed by the University of California, Berkeley (345), and Columbia University (311). In this table, mediating centrality represents the proportion of the connection between all other nodes in the cooperative network, and the value range is between 0 and 1. The higher the value is, the more important the node is.

**Figure 3:**
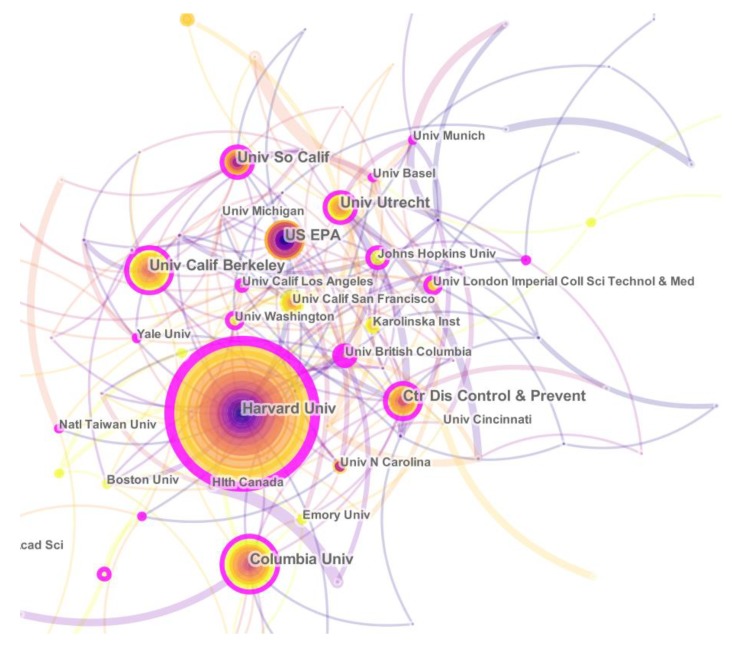
Map of active institutions in the research field from 1999 to 2018. The font size of the name represents the number of articles published by the institution. The purple ring of the circles indicates the core institution. The thickness of the curved connecting line represents the collaborative intensity between the institutions.

In Figure 3, extensive cooperating relationships were observed among institutions. The greater the font size of the name, the more cooperation for the agency. The University of British Columbia, the University of Washington, and Utrecht University had thick purple outer rings, indicating that these nodes have a high degree of betweenness centrality and play an important role in mediating the composition of cooperative networks.

### 3.4. Analysis of Reference Co-Citation

Table 3 is generated on the basis of publications between 1999 and 2018. The top 50 most cited publications in each two years are used to construct a network of references cited in that year. Then, individual networks are synthesized. The synthesized network contains 323,116 references. The network contains 377 nodes and was divided into 11 co-citation clusters. The four largest connected components include 104 nodes, which account for 56.76% of the entire network. The network has a modularity of 0.7346. The average silhouette score of 0.3352 is relatively low, this is mainly because of the numerous small clusters. The first cluster is marked with “#0 household air pollution”, which indicates that the luster is cited by the articles on household air pollution. Eleven clusters differ from one another in the aspect of starting time, time span, or activeness, e.g., the duration of cluster “#0 household air pollution” was the longest, lasting from 2007 to 2017. The cluster “#1 nitrogen dioxide” appeared earlier, however it discontinued around 2009.

### 3.5. Analysis of Keywords and Burst Keywords

According to the keyword co-occurrence analysis, 55 keywords were detected. The keywords with strong citation bursts were explored through CiteSpace, and 24 keywords with the strongest strength in the last decades were identified (Table 4) [21,22]. Strength means citation burst, and it represents the increasing frequency of keywords in the corresponding period. The appearance and duration time of the burst keywords represents the active time for these burst keywords. From 2009 to 2018, the burst key words changed from smoke (‘nicotine’, ‘tobacco smoke’, ‘secondhand smoke’) to fine particulate matter and ambient air pollution. The mechanism of PM2.5 on health focused on inflammation, not oxidation.

## 4. Discussion

### 4.1. General Information

In this study, we found that the literature about air pollution and children showed an increasing trend. From 1999 to 2018, the number of related literatures increased from 498 to 1213. Such a phenomenon is consistent with research focus in recent years. In recent years, the impacts of air pollution on health have attracted world-wide attention because of its big disease burden. Numerous stations have been built to obtain air pollution exposure data, especially for PM_2.5_. Furthermore, many lab studies have been carried out to explore the mechanism of air pollution on health, through the inflammation field.

In this field, 3203 papers were published in the top ten journals, around 20.2% of the total literature. Such results suggested that these journals covered the field about air pollution and children. Academics in this field should submit their papers to these journals. In addition, papers on the relationship between air pollution and children were published in environmental journals, not clinical or children specific journals.

We also found a close cooperation among institutes. In the top ten institutes in this field, the top three institutes were Harvard University (585), the University of California, Berkeley (345), and Columbia University (311). They also have higher mediating centrality, which indicates that these agencies were very active in the cooperation network.

### 4.2. Research Hotspots Analysis

The clusters of the top 50 most cited publications in each two years were converged. And the modularity of the network is 0.7346, which is usually considered to be relatively high, suggesting that the specialties in the impact of air pollution on children are clearly defined in terms of co-citation clusters. We just tried to divide the four active clusters into two categories: exposure (Clusters 0 and 5) and health outcome (Clusters 8 and 9).

Topic 1 (Clusters 0 and 5) demonstrated the exposure types which attracted more attention in recent epidemiological studies. It is well known that air pollution is a risk factor for the population, especially for children [23]. Considering that children spend time both indoors and outdoors, many studies consider both indoor and outdoor exposure [24]. Air pollution has adverse effects on cardiorespiratory effects, including asthma prevalence [25]. Researchers have come to explore the possible role of air pollution exposure in utero and early life. Based on one cohort in British Columbia in 1999 and 2000, they found early life exposure to CO, NO, NO_2_, PM_10_, SO_2_, and black carbon could significantly increase the risk of asthma and early childhood exposure to air pollutants should be avoided [26]. As for Cluster 5, ‘passive smoke’ is more precise than ‘smoke-free legislation’. The ratio of children exposed to passive smoke is highest in the whole population [27] Compared with indoor air pollution, the study about passive smoke attracted less attention based on the duration time.

Topic 2 (Clusters 8 and 9) reflected the health outcomes that researchers focused on in recent years. Autistic disorder (AD) is a serious developmental disorder to which genetic and environmental factors likely contribute [28]. A lot of epidemiological studies provided evidence for the genetic interaction with environmental factors for autism [29]. One cross-section study in California found that ambient air pollutants, such as ozone and nitric oxide, are associated with autism [30]. As for Cluster 9, ‘lung function’ is more precise than ‘early childhood ear infection’. Based on previous studies, the association between ambient air pollution and lung function was positive. Decreased lung function has a link with increased incidence of asthma [31]. Three separate cohorts were used to examine the association between air pollution and children lung function. The results showed that lung function improved with better air quality [32]. We recognized that ‘lung function’ should be paid more attention, compared with ‘autism’.

### 4.3. Burst Keywords

Burst keywords refer to keywords heavily cited by articles over a period of time. Burst keywords are considered another important indicator of research hotspots or emerging trends over time. As seen in Table 4, the evolution of the burst keywords during the past decade shows the continuing progress in impacts of air pollution on children’s health research. As for the exposure index, there is one obvious transition from the smoke-related index (such as nicotine, second-hand smoke) to the ambient air pollution index (fine particulate matter). Fine particulate matter has become one hotspot in china because of its bad air quality [33]. The related mechanism also is explored, and more studies have been carried out about inflammation [34]. As for the study design, there is also one obvious transition from time series to cohort. In current days, most cohort studies are carried out in developed countries with low air pollution exposure, so it is hard for researchers to get an accurate estimation about the impacts of high air pollution exposure on children [35]. The air pollution level in china is still well beyond the WHO recommended air quality criteria (10 μg/m^3^), so there is urgent need for a cohort study in this field in China [36,37,38].

## 5. Conclusions

On the basis of the quantitative analysis of co-citation and burst words analysis, researchers in this field can have a comprehensive understanding of the latest popular topics in the field of air pollution and children’s health. Researchers in this field could carry out more cohort studies and fine particulate matter is one good air pollution index. Household air pollution exposure and children’s lung function should be paid more attention to.

## Figures and Tables

**Table 1:** The top 10 journals of research on air pollution and children.

Rank	Journal	*N* (%)	Country	IF (2018)	Classification
1	ENVIRONMENTAL HEALTH PERSPECTIVES	596(3.73%)	USA	8.049	(1–3)
2	ENVIRONMENTAL RESEARCH	450(2.81%)	USA	5.026	(1,2)
3	SCIENCE OF THE TOTAL ENVIRONMENT	377(2.36%)	NETHERLANDS	5.589	(1)
4	INTERNATIONAL JOURNAL OF ENVIRONMENTAL RESEARCH AND PUBLIC HEALTH	322(2.01%)	SWITZERLAND	2.468	(1,2)
5	ENVIRONMENT INTERNATIONAL	303(1.89%)	USA	7.943	(1)
6	PLOS ONE	255(1.59%)	USA	2.776	(4)
7	INDOOR AIR	242(1.51%)	DENMARK	4.710	(2,5,6)
8	JOURNAL OF ALLERGY AND CLINICAL IMMUNOLOGY	228(1.43%)	USA	14.110	(7,8)
9	ATMOSPHERIC ENVIRONMENT	221(1.38%)	ENGLAND	4.012	(1,9)
10	PEDIATRICS	209(1.31%)	USA	5.401	(10)
11	AMERICAN JOURNAL OF RESPIRATORY AND CRITICAL CARE MEDICINE	199(1.244%)	USA	16.494	(11–13)
12	ENVIRONMENTAL HEALTH	198(1.237%)	ENGLAND	4.430	(1,2)
13	EUROPEAN RESPIRATORY JOURNAL	174(1.087%)	ENGLAND	11.807	(11)
14	NICOTINE TOBACCO RESEARCH	174(1.087%)	ENGLAND	3.786	(2,14)
15	BMC PUBLIC HEALTH	167(1.044%)	ENGLAND	2.567	(2)
16	JOURNAL OF ASTHMA	167(1.044%)	USA	2.081	(11,15)
17	OCCUPATIONAL AND ENVIRONMENTAL MEDICINE	156(0.975%)	ENGLAND	3.556	(2)
18	ENVIRONMENTAL SCIENCE AND POLLUTION RESEARCH	155(0.969%)	GERMANY	2.914	(1)
19	INTERNATIONAL JOURNAL OF HYGIENE AND ENVIRONMENTAL HEALTH	147(0.919%)	GERMANY	4.379	(2,16)
20	JOURNAL OF EXPOSURE SCIENCE AND ENVIRONMENTAL EPIDEMIOLOGY	145(0.906%)	USA	3.025	(1–3)

These literatures were classified by journal citation reports: (1) ENVIRONMENTAL SCIENCES, (2) PUBLIC, ENVIRONMENTAL & OCCUPATIONAL HEALTH, (3) TOXICOLOGY, (4) MULTIDISCIPLINARY SCIENCES, (5) CONSTRUCTION & BUILDING TECHNOLOGY, (6) ENGINEERING, ENVIRONMENTAL, (7) ALLERGY, (9) IMMUNOLOGY, (9) METEOROLOGY & ATMOSPHERIC SCIENCES, (10) PEDIATRICS, (11) RESPIRATORY SYSTEM, (12) CRITICAL CARE MEDICINE, (13) EMERGENCY MEDICINE & CRITICAL CARE, (14) SUBSTANCE ABUSE, (15) ALLERGY, (16) INFECTIOUS DISEASES.

**Table 2:** The top fifteen institutes during the period 1999–2018.

Institution	N	Centrality
Harvard University	585	0.13
University of California, Berkeley	345	0.1
United States Environmental Protection Agency	324	0.02
Columbia University	311	0.08
US Centers for Disease Control and Prevention	279	0.08
Utrecht University	250	0.24
University of Southern California	248	0.11
University of California, San Francisco	237	0.09
University of North Carolina at Chapel Hill	229	0.04
University Washington	223	0.17
Karolinska Institute	213	0.09
Johns Hopkins University	198	0.14
University of British Columbia	178	0.3
University of California Los Angeles	178	0.14
Emory University	172	0.07

**Table 3:** Cluster analysis of literature from 1999–2018.

Cluster ID	Size	From	To	Activeness	Theme
0	64	2007	2017	Active	Household air pollution
1	61	2001	2009	Inactive	Nitrogen dioxide
2	60	1995	2004	Inactive	Preterm delivery
3	29	1994	1999	Inactive	Outdoor air pollution
4	29	2001	2010	Inactive	Pregnancy outcome
5	25	2005	2015	Active	Smoke-free legislation
6	24	1994	2002	Inactive	High endotoxin level
7	22	1995	2001	Inactive	Parental smoking
8	20	2008	2015	Active	Autism spectrum disorder
9	15	2012	2017	Active	Early childhood ear infection
10	10	2002	2006	Inactive	Indoor environmental influence

**Table 4:** The analysis of keywords from 1999 to 2018.

No.	Keywords	Strength	Begin	End
1	Nicotine	26.95	2009	2010
2	Tobacco smoke	31.36	2009	2015
3	Indoor	39.65	2009	2014
4	Quality	33.27	2011	2018
5	Secondhand smoke	34.36	2011	2015
6	Birth weight	24.97	2011	2012
7	Indoor air pollution	61.77	2011	2014
8	Developing country	29.11	2013	2014
9	Particulate air pollution	5.22	2013	2014
10	Intervention	35.84	2013	2014
11	Young children	13.70	2013	2014
12	Cohort	33.90	2013	2018
13	Adult	9.54	2013	2014
14	Impact	59.26	2014	2018
15	China	65.04	2014	2018
16	Polycyclic aromatic hydrocarbon	22.64	2014	2018
17	Outcome	34.96	2015	2016
18	Heavy metal	68.63	2015	2018
19	Long term exposure	35.50	2015	2016
20	Time series	27.16	2015	2016
21	Oxidative stress	27.16	2015	2016
22	Inflammation	27.51	2015	2018
23	Fine particulate matter	63.54	2015	2018
24	Ambient air pollution	34.99	2015	2018

## Supplementary Materials

The following are available online at https://www.mdpi.com/1660-4601/17/4/1277/s1, Table S1: Literature retrieval strategy in the PubMed. Table S2: Literature retrieval strategy in the Web of Science.
