# Supplementary material for: A Bibliometric Analysis of the Impacts of Air Pollution on Children
Source: Int J Environ Res Public Health. 2020 Feb 17;17(4):1277. doi: 10.3390/ijerph17041277 (PMC7068507; doi:10.3390/ijerph17041277)
Supplement: Supplementary file 1 [file ijerph-17-01277-s001.pdf]

Supplemental material

A Bibliometric Analysis of the Impacts of Air Pollution on Children

Table S1 Literature retrieval strategy in the PubMed.

Table S2 Literature retrieval strategy in the Web of Science.

Table S1 Literature retrieval strategy in the PubMed.

Retrieved on 23 June 2019

Search	Query	results
#1	Search "Air Pollution"[Mesh] OR "Air Pollutants"[Mesh] OR "Particulate Matter"[Mesh] OR "Nitrogen Dioxide"[Mesh] OR "Sulfur Dioxide"[Mesh] OR "Ozone"[Mesh] OR "Vehicle Emissions"[Mesh] OR "Carbon monoxide"[Mesh]	156395
#2	Search "ambient air pollution"[Text Word] OR PM2.5[Text Word] OR PM10[Text Word] OR SO2[Text Word] OR O3[Text Word] OR "Carbon monoxide"[Text Word] OR "air quality"[Text Word] OR "Atmospheric pollution"[Text Word] OR "inhalable particles"[Text Word] OR "Inhalable particulate matter"[Text Word]	68304
#3	Search "Infant"[Mesh] OR "Child"[Mesh] OR "Adolescent"[Mesh] OR "Pediatrics"[Mesh]	3435112
#4	Search (#1 OR #2) AND #3 AND ("1999"[Date - Publication] : "2018"[Date - Publication])) AND English[Language]	12156

12156 PMID were extracted from search results

Table S2 Literature retrieval strategy in the Web of Science.

search	Query	results
#1	PMID=(31126003 OR 30766556 OR 30759582 OR 30682747 OR 30677904 OR 30677897 OR ……)	11051
#2	(TS=(Air Pollution OR Air Pollutants OR Particulate Matter OR Nitrogen Dioxide OR Sulfur Dioxide OR Ozone OR Vehicle Emissions OR Carbon monoxide) OR TS=(ambient air pollution OR PM2.5 OR PM10 OR SO2 OR O3 OR Carbon monoxide OR air quality OR Atmospheric pollution OR inhalable particles OR Inhalable particulate matter)) AND language: (English) *Indexes=SCI-EXPANDED, SSCI Timespan=1999-2018*	286,945
#3	(#2 AND TS=(Infant OR Child OR Adolescent OR Pediatrics)) AND language: (English) *Indexes=SCI-EXPANDED, SSCI Timespan=1999-2018*	9,503
#4	(#3 OR #1) AND language: (English) AND DOCUMENT TYPES: (Article OR Letter OR Review) *Indexes=SCI-EXPANDED, SSCI Timespan=1999-2018*	15,999
